# Experimental hyperoxia (O_2_ supersaturation) reveals a gill diffusion limitation of maximum aerobic performance in fish

**DOI:** 10.1098/rsbl.2022.0401

**Published:** 2022-11-02

**Authors:** T. J. McArley, D. Morgenroth, L. A. Zena, A. T. Ekström, E. Sandblom

**Affiliations:** Department of Biological and Environmental Sciences, University of Gothenburg, PO Box 463, 405 30 Gothenburg, Sweden

**Keywords:** hyperoxia, O_2_ supersaturation, aerobic scope, cardiac output, exhaustive exercise, oxygen consumption

## Abstract

Several studies have demonstrated that hyperoxia increases the maximal O_2_ consumption rate (ṀO_2max_) in fish, but exactly how this occurs remains to be explained. Here, we tested the hypothesis that hyperoxia improves arterial oxygenation in rainbow trout during exhaustive exercise. We demonstrate a 35% higher ṀO_2max_ in hyperoxia (200% air saturation) relative to normoxia, which was achieved through a combined 15% increase in cardiac output due to elevated peak heart rate, and a 19% increase of the arterial–venous (A-V) O_2_ content difference. While arterial O_2_ partial pressure (PaO_2_) and O_2_ saturation of haemoglobin declined post-exhaustive exercise in normoxia, this did not occur in hyperoxia. This protective effect of hyperoxia on arterial oxygenation led to a 22% higher arterial O_2_ content post-exhaustive exercise, thereby allowing a higher A-V O_2_ content difference. These findings indicate that ṀO_2max_ is gill diffusion limited in exhaustively exercised rainbow trout. Moreover, as previous studies in salmonids have demonstrated collapsing PaO_2_ in normoxia at maximal swimming speed and at acutely high temperatures, a diffusion limitation may constrain ṀO_2_ in other situations eliciting peak metabolic demand. These findings, along with the fact that hyperoxia increases ṀO_2max_ in several other fishes, suggest that gill diffusion limitations of ṀO_2max_ may be widespread in fishes.

## 1. Introduction

Several studies have demonstrated that hyperoxia (O_2_ supersaturation) increases the maximum rate of O_2_ consumption (ṀO_2max_) and aerobic metabolic scope (the difference between ṀO_2max_ and resting ṀO_2_) of fish [1–3]. In rainbow trout (*Oncorhynchus mykiss* (Walbaum 1792)), we recently linked this improved aerobic capacity in hyperoxia to elevated blood pumping capacity (higher cardiac output) of the heart, possibly due to an enhanced myocardial O_2_ supply resulting from elevated partial pressure of O_2_ in the venous blood (PvO_2_) returning to the heart [1]. An anomalous finding of our previous study, however, was that, despite hyperoxia exposed fish having lower haemoglobin levels and higher PvO_2,_ the arterial–venous (A-V) O_2_ content difference (the amount of O_2_ extracted from blood at the tissues) tended to be higher in hyperoxia than normoxia. Lower haemoglobin tends to reduce arterial O_2_ content (CaO_2_), while higher PvO_2_ tends to increase venous O_2_ content, which theoretically should reduce the A-V O_2_ content difference. A possible explanation for this finding could be that elevated arterial PO_2_ (PaO_2_) in hyperoxia—a common response of fish at rest [4]—has a protective effect on O_2_ saturation of haemoglobin (HbO_2_), thereby promoting higher CaO_2_. To assess ṀO_2max_ in fish, it is common to chase a fish to exhaustion over several minutes and then assign ṀO_2max_ to the highest rate of O_2_ consumption measured immediately following exercise cessation [5]. Several studies in rainbow trout have demonstrated that PaO_2_ and HbO_2_ saturation can decline immediately following exhaustive exercise in normoxia [6–8]. Thus, when using the chasing approach to elicit ṀO_2max_, a window may exist immediately following exhaustive exercise where a higher O_2_ diffusion gradient between hyperoxic water and deoxygenated blood entering the gills could offset declines in PaO_2_ and HbO_2_ saturation that occur in normoxia. This proposed protective effect of hyperoxia on arterial oxygenation following exhaustive exercise could increase CaO_2_, thereby leading to an increased A-V O_2_ content difference, which in combination with higher cardiac output (blood flow to the tissues) would ultimately elevate ṀO_2max_. This is neatly summarized by the Fick equation, where ṀO_2_ is the product of cardiac output multiplied by the A-V O_2_ content difference.

Here, using rainbow trout as a model species, we tested the hypothesis that higher ṀO_2max_ and aerobic scope of fish in hyperoxia results from a combination of increased cardiac output and protection of arterial blood oxygenation immediately following exhaustive exercise. We made three predictions: (i) hyperoxia would offset declines in PaO_2_ and arterial HbO_2_ saturation that occur following exhaustive exercise in normoxia; (ii) relative to normoxia, hyperoxia would increase CaO_2_ following exhaustive exercise and promote a higher A-V O_2_ content difference and (iii) ṀO_2max_ would ultimately be higher under hyperoxia relative to normoxia due to a combination of elevated A-V O_2_ content difference and elevated cardiac output. To assess these predictions, we performed simultaneous measurements of MO_2max_, cardiac output and arterial blood oxygenation in rainbow trout exhaustively exercised under normoxic and hyperoxic (200% air saturation) water O_2_ levels.

## 2. Methods

### (a) Animals and holding facilities

The rainbow trout (mass: 1346 ± 34.12 g) examined in this study were obtained from a commercial hatchery (Vänneåns Fiskodling AB, Halland, Sweden). Prior to experimentation, they were held in 600 l tanks for at least four weeks of laboratory acclimation. These tanks were supplied with air saturated, recirculated fresh water at a constant temperature of approximately 10°C (absolute range: 9.7–10.3°C) and maintained under a 12 h : 12 h light : dark photoperiod. Fish were fed pellets (7 mm, Protec Trout pellets, Skretting, Norway) twice a week until the start of the experiments, but food was always withheld for 3 days prior to experimentation.

### (b) Experimental protocol

This study followed a paired design, where all individuals were subjected to the same experimental manipulations under O_2_ conditions of normoxia (approx. 100% air saturation or approximately 21 kPa) and hyperoxia (approx. 200% air saturation or approximately 41 kPa) at 9.8 ± 0.03°C (see electronic supplementary material, figure S1 for a visual outline). The mean PaO_2_ inside the respirometers was 20.1 ± 0.05 kPa and 43.5 ± 0.14 kPa in the normoxia and hyperoxia treatments, respectively. An individual protocol was carried out over 3 days. On day 1, fish were anaesthetized (150 mg l^−1^ MS-222 buffered with 300 mg l^−1^ NaHCO_3_) and fitted with a ventral aortic blood flow probe (2.5 mm Transonic L type; Transonic Systems, Ithaca, NY) following the method outlined in Morgenroth *et al*. [9]. A PE50 cannula was also placed in the dorsal aorta to allow sampling of post-branchial arterial blood. Following surgery, the fish was placed in a 10 l respirometer held in a 120 l experimental tank and allowed to recover overnight under conditions of normoxia. On day 2 and day 3, fish were exhaustively exercised under either normoxia or hyperoxia. Seven fish were exposed to normoxia on day 2 and hyperoxia on day 3, and six fish were exposed to hyperoxia on day 2 and normoxia on day 3. For the hyperoxia treatment, the water-supplying respirometers was bubbled with O_2_ to establish 200% air saturation (42 kPa) approximately 2 h prior to exhaustive exercise. A resting blood sample (approx. 200 µl) was first taken, whereafter the fish was removed from the respirometer and exhaustively exercised in a circular 50 l tank by manual chasing (5 min) under its respective O_2_ treatment condition. It was then returned to the respirometer for assessment of maximal post-exhaustive exercise cardiorespiratory function and a post-exhaustive exercise blood sample was taken. For the hyperoxia treatment, 200% air saturation (42 kPa) was maintained for approximately 2 h following exhaustive exercise. Between day 2 and day 3 chasing episodes, the fish recovered overnight under normoxia.

### (c) Parameters assessed

Mass-specific O_2_ consumption rate (ṀO_2_), cardiac output, heart rate, stroke volume and the A-V O_2_ content difference (Fick estimated) were recorded and assessed using the same equipment, protocols and calculations outlined in McArley *et al*. [1]. Resting cardiorespiratory variables were taken as the mean of three 6–8 min measurement periods immediately prior to exhaustive exercise. ṀO_2max_ was defined as the highest 2 min period of ṀO_2_ recorded, which occurred within 8 min of exhaustive exercise cessation in all fish. Maximal cardiac function (cardiac output, heart rate and stroke volume) and maximal A-V O_2_ content difference were tied to the same measurement time point as ṀO_2max_. From the dorsal aortic blood samples, PaO_2_ was assessed following the protocol outlined for venous blood in McArley *et al*. [1], and blood O_2_ content was assessed following the protocol outlined in Brijs *et al*. [10]. Physically dissolved plasma O_2_ content and haemoglobin-bound O_2_ content were calculated using the method outlined in Milligan & Wood [6]. Finally, blood haemoglobin and haematocrit were assessed following the method outlined in McArley *et al*. [1].

### (d) Statistics

All statistical analysis were performed using GraphPad Prism (version 9.3.1), with significance set at *p* < 0.05. Resting and maximal cardiorespiratory and blood parameters were compared between normoxia and hyperoxia using paired *t*-tests. The normality of residuals assumption was met for all comparisons as assessed by a Shapiro–Wilk test. Detailed statistical outcomes are reported in the electronic supplementary material, table S1.

## 3. Results and discussion

**Figure 1.**
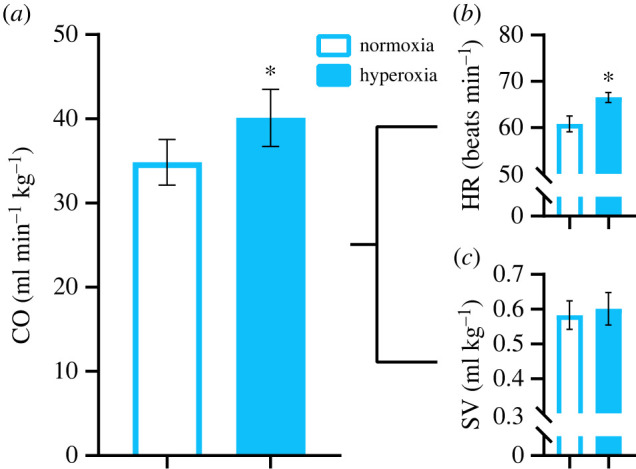
Maximal post-exhaustive exercise cardiac performance in rainbow trout under normoxia or hyperoxia (200% air saturation). (*a*) Cardiac output (CO), (*b*) heart rate (HR), (*c*) stroke volume (SV). Values are means ± s.e.m. (*N* = 11–12). Asterisks show significant (*p* < 0.05) differences (see electronic supplementary material, table S1 for detailed statistical results).

**Figure 2.**
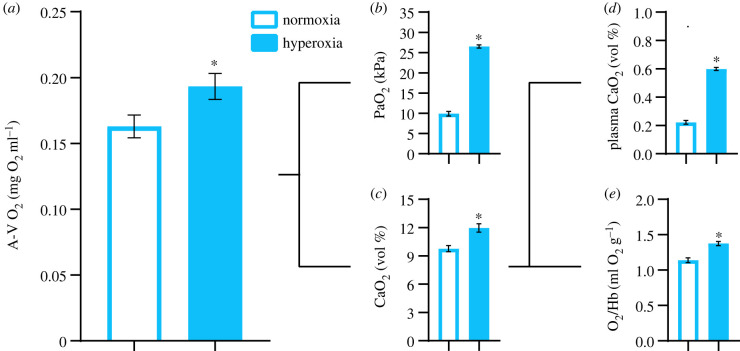
Post-exhaustive exercise blood oxygenation in rainbow trout under normoxia or hyperoxia (200% air saturation). (*a*) Arterial–venous O_2_ content difference (A-V O_2_), (*b*) PaO_2_ in dorsal aortic blood (PaO_2_), (*c*) total O_2_ content of dorsal aortic blood, (*d*) content of physically dissolved O_2_ in dorsal aortic blood plasma (plasma CaO_2_) and (*e*) haemoglobin-bound O_2_ (O_2_/Hb). Values are means ± s.e.m. (*N* = 11–13). Asterisks show significant (*p* < 0.05) differences (see electronic supplementary material, table S1 for detailed statistical results).

ṀO_2max_ was 35% higher under hyperoxia than normoxia (table 1). This increase was similar to our previous study, where ṀO_2max_ increased by 33% under hyperoxia [1]. As resting ṀO_2_ was essentially identical between O_2_ levels, aerobic scope was also elevated in hyperoxia (table 1). Here, the higher ṀO_2max_ and aerobic scope in hyperoxia was driven by a combination of a 15% increase in cardiac output (figure 1*a*) and a 19% increase in A-V O_2_ content difference (figure 2*a*). Somewhat contrasting our previous study, where there was a trend (albeit not quite significant) for elevated stroke volume post-exhaustive exercise, elevated cardiac output with hyperoxia in the current study resulted mostly from increased heart rate (figure 1*b,c*). Furthermore, the advantage of hyperoxia to cardiac output was somewhat less pronounced than the 20% increase we observed in our previous study [1]. A possible explanation for these somewhat conflicting findings is that blood haematocrit levels were identical between hyperoxia and normoxia in the current study (table 1), whereas they were significantly reduced with hyperoxia in our previous study. This difference may have been due to the shorter duration of hyperoxia exposure prior to blood sampling used in the current study (approx. 2 h here versus approximately 22 h previously). Experimentally elevated haematocrit, which increases blood viscosity [11], has previously been shown to reduce maximum cardiac output through limiting maximum stroke volume in swimming rainbow trout [12]. Thus, the apparent advantage of hyperoxia to stroke volume we previously observed may have been, at least in part, the result of lower haematocrit and reduced blood viscosity rather than a direct effect of an enhanced heart O_2_ supply improving contractility.

**Table 1.** Cardiorespiratory parameters, blood oxygenation and haematology of rainbow trout under normoxia and hyperoxia (200% air saturation). Values are means with s.e.m. in parenthesis (*N* = 10–13). Asterisks show significant differences (*p* < 0.05) (see electronic supplementary material, table S1 for detailed statistical results).

	resting (pre-exhaustive exercise)		maximum (post-exhaustive exercise)	
	normoxia	hyperoxia	normoxia	hyperoxia
ṀO_2_ (mg O_2_ kg^−1^ h^−1^)	50.44 (2.6)	48.86 (3.1)	322.3 (8.7)	435.6 (21.4)*
aerobic scope (mg O_2_ kg^−1^ h^−1^)			271.9 (8.6)	386.8 (19.7)*
cardiac output (ml min^−1^ kg^−1^)	16 (1.3)	14.2 (1.3)	34.8 (2.7)	40.1 (1.6)*
heart rate (beats min^−1^)	47.2 (2.8)	44 (2.5)	60.8 (1.7)	66.5 (1.1)*
stroke volume (ml kg^−1^)	0.36 (0.04)	0.34 (0.03)	0.58 (0.04)	0.60 (0.05)
A-V O_2_ content (mg O_2_ ml^−1^)	0.055 (0.002)	0.062 (0.004)	0.16 (0.01)	0.19 (0.01)*
PaO_2_ (kPa)	15.8 (0.4)	26.2 (0.8)*	9.9 (0.6)	26.5 (0.4)*
CaO_2_ (vol %)	10.2 (0.2)	10.9 (0.4)*	9.8 (0.3)	12 (0.4)*
plasma CaO_2_ (vol %)	0.39 (0.01)	0.65 (0.02)*	0.22 (0.01)	0.60 (0.01)*
haemoglobin-bound O_2_ (ml g Hb^−1^)	1.34 (0.03)	1.41 (0.03)	1.14 (0.03)	1.38 (0.03)*
haematocrit (%)	24.8 (0.7)	24.2 (0.7)	31.4 (0.9)	30.6 (0.9)
haemoglobin (g l^−1^)	73.8 (2.3)	72.5 (1.6)	83.7 (1.7)	82.3 (2.3)
ṀO_2_ = mass-specific O_2_ consumption rate; A-V O_2_ content = arterial–venous O_2_ content difference (estimated by the Fick equation); PaO_2_ = partial pressure of O_2_ in dorsal aortic blood; CaO_2_=O_2_ content of dorsal aortic blood; plasma CaO_2_ = content of physically dissolved O_2_ in the blood plasma

The elevated A-V O_2_ content difference in hyperoxia was due to a 22% higher CaO_2_ (figure 2*c*). This resulted from a combination of increased physically dissolved O_2_ in the blood plasma and, most importantly, increased HbO_2_ saturation (haemoglobin-bound O_2_) (figure 2*d*,*e*). Both these effects almost certainly resulted from the substantially greater PaO_2_ in hyperoxia relative to normoxia immediately following exhaustive exercise (figure 2*b*). In normoxia, PaO_2_ fell considerably from 15.8 ± 0.4 kPa at rest to 9.9 ± 0.6 kPa following exhaustive exercise, as has previously been demonstrated after exhaustive exercise in rainbow trout [6–8]. This collapse in PaO_2_ did not occur in hyperoxia, where post-exhaustive exercise PaO_2_ (26.5 ± 0.4 kPa) was maintained at the same level as resting PaO_2_ (26.2 ± 0.8 kPa). Thus, in line with our predictions, through a protective effect on arterial oxygenation and HbO_2_ saturation following exhaustive exercise, hyperoxia increased CaO_2_ and drove a higher A-V O_2_ content difference.

Our findings suggest, at least following exhaustive exercise, that ṀO_2max_ is gill diffusion limited in rainbow trout. Hyperoxia also increases ṀO_2max_ following exhaustive exercise in European perch (*Perca fluvialitis*) and triplefin fishes [2,3], suggesting a diffusion limitation of ṀO_2max_ may be widespread in fishes. Furthermore, maximum routine ṀO_2_ is expanded in hyperoxia at temperatures approaching critical thermal limits in European perch [3] and rainbow trout [13] indicating a diffusion limitation of ṀO_2_ may also occur at other times of peak metabolic demand. The cause of the collapse in arterial PaO_2_ following exhaustive exercise is unknown. One possibility is that gill ventilation is insufficient to allow optimal transfer of O_2_ from water to blood at the gills during periods of peak metabolic demand. Indeed, in rainbow trout, Primmet *et al*. [8] noted a reduction in gill ventilation frequency immediately following exhaustive exercise at the same point PaO_2_ collapsed. Suboptimal gill ventilation could be exacerbated when ṀO_2_ is measured in static respirometers, as in the current study, where water flow across the gills is reliant on buccal pumping. Rainbow trout and other highly aerobic, athletic fishes, however, can transition to ram ventilation as O_2_ demand increases with swimming speed [14]. If inadequate water flow across the gills while buccal pumping is the primary cause of collapsing PaO_2_ in normoxia, then it would be predicted this phenomenon does not occur in swimming fish that can ram ventilate. Indeed, while some studies on salmonids have shown PaO_2_ to remain stable with increasing swimming speed [15,16], several others have demonstrated a decline in PaO_2_ in line with that observed post-exhaustive exercise [12,16–22]. Adding further complexity is the fact that ṀO_2max_ did not increase with hyperoxia in swimming rainbow trout [23]. We believe the balance of evidence suggests that a decline in PaO_2_ in normoxia occurs both post-exhaustive exercise and at maximal swimming speeds in salmonids. Furthermore, this also appears to occur with acute thermal ramping [24,25]. While hyperoxia will generally offset this decline, the degree to which ṀO_2max_ increases with hyperoxia likely depends on whether or not it also offsets O_2_ desaturation of haemoglobin. If, despite arterial hypoxemia due to collapsing PaO_2_, HbO_2_ saturation does not decline in normoxia, hyperoxia will only modestly increase CaO_2_ and therefore be unlikely to meaningfully increase the A-V O_2_ content difference. In such a scenario, any increase in ṀO_2max_ would then become more dependent on whether hyperoxia also increases maximal cardiac output. As the extent of O_2_ desaturation of haemoglobin in normoxia is likely to play an important role in dictating the extent to which hyperoxia can boost ṀO_2max_, it may be that the largest effects of hyperoxia will be observed under conditions that reduce HbO_2_ affinity. The severe blood acidosis following exhaustive exercise in fish and potentially associated reductions in HbO_2_ binding affinity due to Root and Bohr effects [6], for example, could amplify the extent to which hyperoxia increases ṀO_2max_.

Increasingly, naturally occurring hyperoxia is being touted as an environmental phenomenon that can improve the resilience of aquatic ectotherms, including fish, to extreme heat waves associated with climate change [4,26,27]. Moreover, there is growing interest in using hyperoxia as an experimental tool to examine whether O_2_ capacity limitations set constraints on whole animal performance in fish facing environmental warming [4,28]. The primary advantage of hyperoxia to fish, as seen here in rainbow trout and previously in rainbow trout and several other species, is an expansion of ṀO_2max_ and aerobic scope. This could potentially mitigate constraints on aerobic performance that would otherwise occur under normoxia when fish face acutely rising temperatures in shallow aquatic habitats, where hyperoxia and acute warming can often co-occur. Moreover, in some cases, hyperoxia can increase the absolute thermal limits of fish [4,13,27,29]. Here, we reveal that expanded aerobic capacity in hyperoxia can result not only from increased cardiac performance but also from a protective effect on arterial blood oxygenation. If the apparent gill diffusion limitation of ṀO_2max_ observed in normoxia is common among fishes, expanded aerobic performance in hyperoxia could be widespread, suggesting many species could potentially benefit from environmental hyperoxia.

## Data Availability

Manuscript data (individual data values for all parameters reported in figures and tables) are available as electronic supplementary material (MS data_Experimental hyperoxia (O_2_ supersaturation) reveals a gill diffusion limitation of maximum aerobic performance in fish) [30].
